# Palliative Effect of Resveratrol against Nanosized Iron Oxide-Induced Oxidative Stress and Steroidogenesis-Related Genes Dysregulation in Testicular Tissue of Adult Male Rats

**DOI:** 10.3390/ijerph19138171

**Published:** 2022-07-04

**Authors:** Mona M. Ahmed, Mohamed M. A. Hussein, Taisir Saber, Yasmina M. Abd-Elhakim

**Affiliations:** 1 Department of Forensic Medicine and Toxicology, Faculty of Veterinary Medicine, Zagazig University, Zagazig 4511, Egypt; monaya@zu.edu.eg; 2 Department of Biochemistry, Faculty of Veterinary Medicine, Zagazig University, Zagazig 4511, Egypt; hamza_vet@zu.edu.eg; 3 Department of Clinical Laboratory Sciences, College of Applied Medical Sciences, Taif University, P.O. Box 11099, Taif 21944, Saudi Arabia; t.saber@tu.edu.sa

**Keywords:** iron oxide nanoparticles, resveratrol, male sex hormones, oxidative stress, steroidogenesis-related genes

## Abstract

The nano-sized iron oxide (Fe_2_O_3_-NPs) is one of the most used engineered nanomaterials worldwide. This study investigated the efficacy of natural polyphenol resveratrol (RSV) (20 mg/kg b.wt, orally once daily) to alleviate the impaired sperm quality and testicular injury resulting from Fe_2_O_3_-NPs exposure (3.5 or 7 mg/kg b.wt, intraperitoneally once a week) for eight weeks. Spermiograms, sexual hormonal levels, oxidative stress indicators, and lipid peroxidation biomarker were assessed. Moreover, the steroidogenesis-related genes mRNA expressions were evaluated. The results showed that RSV substantially rescued Fe_2_O_3_-NPs-mediated sperm defects. Additionally, the Fe_2_O_3_-NPs-induced depressing effects on sperm motility and viability were markedly counteracted by RSV. Moreover, RSV significantly restored Fe_2_O_3_-NPs-induced depletion of testosterone, follicle-stimulated hormone, luteinizing hormone, and testicular antioxidant enzymes but reduced malondialdehyde content. Furthermore, the Fe_2_O_3_-NPs-induced downregulation of steroidogenesis-related genes (3 β-HSD, 17 β-HSD, and Nr5A1) was significantly counteracted in the testicular tissue of RSV-treated rats. These findings concluded that RSV could limit the Fe_2_O_3_-NPs-induced reduced sperm quality and testicular injury most likely via their antioxidant activity and steroidogenesis-related gene expression modulation.

## 1. Introduction

Incorporating engineered nanomaterials into nanotechnology applications has reached far across various fields, contributing to substantial nanoparticle (NPs) emissions in the surrounding environment [1,2]. The nanosized iron oxide (Fe_2_O_3_-NPs) is classified as one of Europe’s most used engineered nanomaterials for coating products, masts, fillers, plasters, non-metal surface treatment products, clay modeling products, and metal surface therapy products [3]. Fe_2_O_3_-NPs is also significant for its extensive uses, such as magnetic resonance imaging (MRI) [4], inducing magnetic hyperthermia for cancer treatment [5], carriers for targeted drug delivery [6], and tissue repair via soldering or welding [7].

Despite the broad applications of Fe_2_O_3_-NPs, some in vitro and in vivo studies have reported its toxic effects, including cytotoxicity [8], genotoxicity [9], hepatotoxicity [10], neurotoxicity [11], and pulmonary toxicity [12]. Oxidative stress has been implicated as a central mechanism for Fe_2_O_3_-NPs-induced injuries [13]. Moreover, the contribution of the iron ions released from Fe_2_O_3_-NPs in Fenton’s reaction resulting in oxidative damage has been confirmed [14,15,16].

Some recent studies showed that Fe_2_O_3_-NPs are harmful to sperm and testicular tissue. For instance, Sundarraj et al. [15] verified the testicular toxicity in mice following Fe_2_O_3_-NPs (25 and 50 mg/kg) intraperitoneal administration for four weeks. Furthermore, Sundarraj et al. [17] revealed that the oral dosing of 25 and 50 mg/kg b.wt Fe_2_O_3_-NPs in mice for 30 consecutive days adversely affected the prostate gland and seminal vesicle Additionally, the intraperitoneal dosing of Fe_2_O_3_-NPs (300 mg/kg b.wt) for four days induced a substantial decrease in sperm characteristics, including motility, spermatogonia, primary sperm cells, spermatids, Leydig cells, Sertoli, total tubular length, and the volumes of interstitial testicular tissue [18]. However, the underlying mechanisms of Fe_2_O_3_-NPs-induced testicular toxicity still need further investigation.

In this period, an increasing interest is directed to using natural supplements as therapeutic or protective interventions for male fertility disorders [19,20,21,22,23]. Resveratrol (RSV) is a natural phytoalexin antioxidant found in numerous plants, including grapes, blueberries, and peanuts [24,25]. RSV has attracted more attention due to its various biological activities, including antioxidative, anti-inflammatory, cytoprotective, and antiaging effects [26,27,28,29,30]. The RSV antioxidant effect is considered the fundamental mechanism of its health benefits [31]. Moreover, RSV showed potent therapeutic roles against various diseases, including respiratory viral infections [32], Alzheimer’s disease [33], diabetes mellitus [34], and renal diseases [35]. Recent in vivo and in vitro studies have shown that RSV protects sperm cells against lipid peroxidation and augments sperm viability and mitochondrial membrane potential [36,37,38]. Yet, to our knowledge, its ameliorative potentials against Fe_2_O_3_-NPs-induced testicular damage have not been investigated.

Hence, we hypothesized that RSV could offer protection against Fe_2_O_3_-NPs-induced reduced sperm quality and testicular injury in rats. To test this hypothesis, semen assessment, male sex hormones, testicular antioxidants enzymes, and lipid peroxidation indicators were assessed. Additionally, the expression of genes associated with steroidogenesis was studied in order to determine the probable mechanisms behind it.

## 2. Materials and Methods

### 2.1. Chemicals

**Figure 1:**
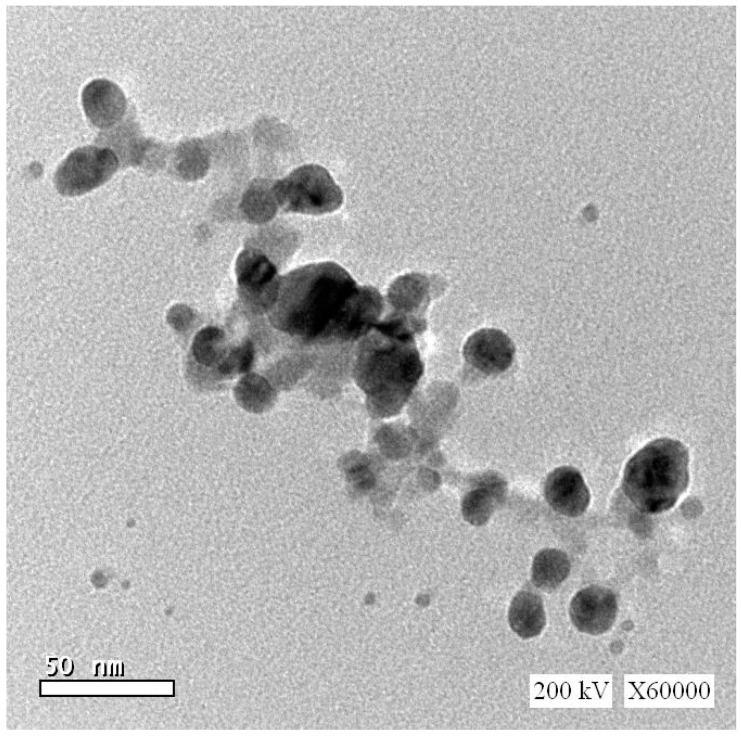
Transmission electron microscopy diffraction patterns for iron oxide nanoparticles.

Fe_2_O_3_ nanopowder (Fe_2_O_3_-NPs, CAS registry number 1309-37-1, catalog number 544884, 99.5% purity, 0.05–0.24 m^2^/kg surface area, <50 nm diameter), RSV (CAS registry number 501-36-0, purity ≥ 99%), epinephrine, reduced glutathione, NADPH, and 5,5 dithiobis 2-nitrobenzoic acid (DTNB) were attained from Sigma-Aldrich (St. Louis, MO, USA). The Fe_2_O_3_-NPs transmission electron microscopy diffraction pattern is shown in Figure 1. All other reagents were obtained from the El-Nasr Company (Cairo, Egypt).

### 2.2. Animals and Experimental Design

Adult male Wistar albino rats (9 weeks old, 0.15–0.16 kg) were kindly obtained from the Animal House at the Faculty of Veterinary Medicine, Zagazig University, Sharkia, Egypt. They were maintained under standard laboratory conditions with free access and water in standard cages with 12 h/12 h dark-light cycles. The experiments were carried out in line with the Ethics Committee of the Faculty of Veterinary Medicine, Zagazig University. After two weeks of acclimation, fifty-four rats were arbitrarily allotted into six groups (9 rats/group). The control group rats received normal saline. The RSV-treated group of rats were orally given 20 mg RSV/kg b.wt once daily. The low and high Fe_2_O_3_-NPs-exposed groups of rats were, respectively, given intraperitoneal (IP) injections of 3.5 and 7 mg/kg b.wt Fe_2_O_3_-NPs dissolved in physiological saline, once a week. The RSV+low Fe_2_O_3_-NPs-exposed group of rats were concurrently treated with RSV and a low dose of Fe_2_O_3_-NPs at the abovementioned route and dose. The RSV+high Fe_2_O_3_-NPs group of rats were treated with RSV and a high dose of Fe_2_O_3_-NPs at the aforementioned dose and route. The experiment extended for eight weeks to cover the duration of spermatogenesis in rats, ranging from 56–60 days [39]. The protocol was permitted by the Institutional Animal Care and Use Committee of Zagazig University (IACUC), Egypt, with approval number (ZU-IACUC/2/F/44/2021). All efforts were made to handle the rats humanely and abide by ethical rules.

### 2.3. The Dose and Route Selection of RSV and Fe_2_O_3_-NPs

The selected RSV oral dose (20 mg RSV/kg b.wt ) has been reported to counteract the testicular toxicity of several environmental contaminants like cadmium [40] and cypermethrin [41]. Additionally, the oral dose of 20 mg RSV/kg b.wt enhanced the rats’ spermatogenesis after 2, 5-hexanedione exposure [42] and is safe in rats [43].

The IP route was selected as a route of administration based on the toxicokinetic studies that confirmed the invasion of Fe_2_O_3_-NPs in different body organs and testis following the intraperitoneal administration [44]. Moreover, comparable toxicological outcomes have been gained through other routes of administration of Fe_2_O_3_-NPs. For instance, Fe_2_O_3_-NPs have been shown to cross the blood–brain barrier, inducing neurotoxic impacts after oral [45], inhalation [46], and intraperitoneal [47] administration. Additionally, Fe_2_O_3_-NPs displayed a lack of genotoxicity following oral [9], IP [48], and intravenous routes [49]. In addition, the IP injection is a common route for administering Fe_2_O_3_-NPs into the body during biomedical applications [50]. Regarding Fe_2_O_3_-NPs doses selection, Prodan et al. [51] found that the IP injection of Fe_2_O_3_-NPs up to 3.7 mL/kg (equivalent to nearly 3.5 mg/kg b.wt) in Brown Norway rats did not induce significant changes in the liver, spleen, or lungs morphology. Nevertheless, the effects of the earlier dose on the testes have not been investigated. Hence, herein, the dose of 3.5 mg/kg b.wt was tested as a low dose and its double (7 mg/kg b.wt) as the high dose.

### 2.4. Blood and Tissue Sampling

All rats were fasted overnight, weighed, and blood samples were collected in non-heparinized tubes at the end of the trial using a retro-orbital plexus site. The serum samples were separated by centrifugation at 1075× *g* for 10 min and stored at −20 °C for further hormonal analysis. After rats were anesthetized by an intramuscular injection of xylazine (5 mg/kg b.wt) and ketamine hydrochloride (50 mg/kg b.wt); euthanized by cervical dislocation; and the testes were harvested, cleaned of surrounding tissues, and weighed with an electronic analytical balance. The gonadosomatic index was calculated according to the following equation: (The average of the right and left testicle weights (g)/the final body weight (g)) × 100 [52]. One side of rat testes was necropsied then directly transmitted in liquid nitrogen to be kept at −80 °C for gene expression. One gram of other-sided ones was homogenized using a tissue homogenizer (Potter–Elvehjem, Thomas Scientific, Swedesboro, NJ, USA) in chilled potassium chloride followed by centrifugation at 3000 rpm for 10 min at 4 °C to collect supernatant that was used to assess the antioxidant status of testicular tissue.

### 2.5. Semen Analysis

The epididymal content of each rat in different experimental groups was obtained by directly cutting the caudal epididymis and smoothly pressing it in a clean petri dish containing 2 mL of pre-warmed physiological saline solution and incubating it at for 30 min 37 °C for liquefaction. The sperm progressive motility was evaluated according to Slott et al. [53] method. One drop of the aforementioned prepared solution was placed on a pre-warmed slide with 80-µm-deep chambers. Then, a high-power light microscope was used to measure the motile spermatozoa percentage in numerous microscopic fields at 400× magnification. Using a hemocytometer, total sperm count was determined in semen diluted with physiological saline at 1:4 with 5 drops of formalin (40%) solution. The total sperm count was calculated as the sum of counted spermatozoa in five secondary square × dilution rate × 2500 and expressed as the total number of spermatozoa/mL [54]. Sperm viability and abnormalities were determined according to the protocol of Filler [55]. A drop of the epididymal content of each rat was mixed with the same amount of eosin–nigrosin stain and spread on grease-free clean slides for the microscopical examination of anomalies in the tail, neck/middle part, and head.

### 2.6. Hormonal Assay

Following the guidelines of manufacturer’s protocols, testosterone (TES), follicle-stimulating hormone (FSH), and luteinizing hormone (LH) were assessed via rat-specific enzyme-linked immunosorbent assay (ELISA) kits no. MBS282195, MBS2502190, and MBS764675, respectively (MyBioSource, San Diego, CA, USA), following the manufacturer’s instructions.

### 2.7. Testicular Oxidative/Antioxidant Status

In testes homogenate, the superoxide dismutase (SOD), catalase (CAT), glutathione peroxidase (GPx), reduced glutathione (GSH), and malondialdehyde (MDA) levels were determined using kits reagents of Bio-diagnostic Co., Giza, Egypt (Catalogue no. SD 25 21, CA 25 17, GP 2524, GR 25 11, and MD 25 29, respectively) following the methods applied by Nishikimi et al. [56], Aebi [57], Paglia and Valentine [58], Beutler [59], and Ohkawa et al. [60], respectively.

### 2.8. Extraction of Total RNA and Real Time-PCR

The total RNA of the testes samples was isolated by Rneasy mini kit (Qiagen) in line with the manufacturer’s protocol guidelines and as previously described [61]. The high quality of RNA samples was tested by 1% gel electrophoresis, and the purity of RNA samples was determined by obtaining the optical density at 260 and 280 nm. Then, 2 µg of total RNA was used to produce cDNA using the RevertAid H minus Reverse Transcriptase kit (Catalog number: EP0451, Thermo Scientific, Waltham, MA, USA). Real-time quantitative PCR was performed by StepOnePlus system (Applied Biosystem, Foster City, CA, USA), where 25 µL PCR reaction mixture was prepared using cDNA (150 ng), forward primer (1 µm), reverse primer (1 µm), Maxima SYBR Green (Catalog number: K0221, Thermo Scientific, Waltham, MA, USA) (12.5 µL), and Rnase free water (Catalog number: 129112, Qiagen Inc., Valencia, CA, USA) (till 25 µL). Rotor-Gene Q2 plex (Qiagen Inc., Valencia, CA, USA) was used to amplify the reaction. The primer sequence used for 3 beta-hydroxysteroid dehydrogenases (*3β-HSD*), 17-beta hydroxysteroid dehydrogenase (*17β-HSD*), and nuclear Receptor Subfamily 5 Group A Member 1 (*Nr5A1*), the PCR amplification condition, and product length are presented in Table 1. *Β-actin* was used as a housekeeping gene. The 2^−ΔΔCt^ method was employed to compute each transcript’s relative quantity in 3 replicates. The period threshold values for the objective genes have been determined concerning the level of *β-actin* mRNA [62].

### 2.9. Statistical Analysis

Data were checked for the normality and homogeneity of variances using the Kolmogorov–Smirnov test and Levene’s test, respectively. Where normality assumptions were met, data were subjected to one-way analysis of variance (ANOVA) to statistically determine the variation between groups followed by Tukey’s multiple range post hoc test for pairwise comparisons. The data have been shown as means ±SD for each group. Mean differences were taken into account at *p* < 0.05. Moreover, the Graphpad computer program was used for regression analysis and data collection (ISI Software, Philadelphia, PA, USA).

## 3. Results

### 3.1. Effect of RSV and/or Fe_2_O_3_-NPs on the Bodyweight Change and Gonadosomatic Index

**Figure 2:**
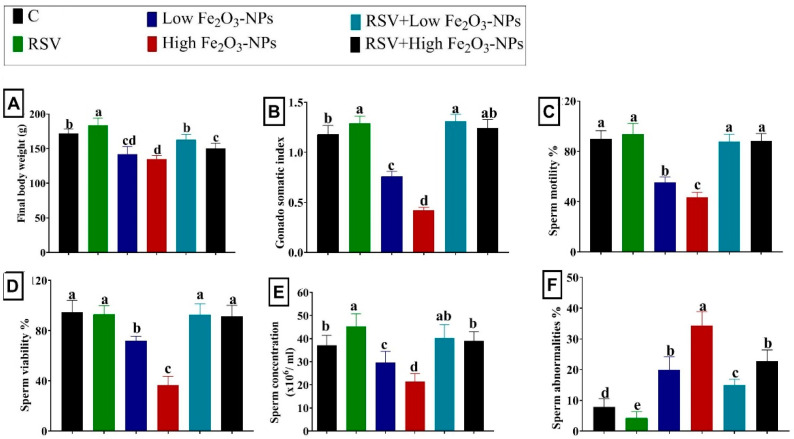
Effect of resveratrol (RSV; 20 mg/kg b.wt) and/or iron oxide nanoparticles (Fe_2_O_3_-NPs 3.5 or 7 mg/kg b.wt) administration for eight weeks on the final body weight (**A**); gonadosomatic index (**B**); sperm motility (**C**); viability (**D**); concentration (**E**); and abnormalities (**F**) of male rats. Data expressed as mean ± SD, *n* = 9 for each group. Different letters (a, b, c, and d) on columns indicate statistically significant differences at *p* < 0.05 between the different experimental groups.

Figure 2A,B showed the final body weight and gonadosomatic index variations in rats who received RSV and/or low or high doses of Fe_2_O_3_-NPs for eight weeks. Initially, the RSV-treated rats increased (*p* < 0.001) in final body weight and gonadosomatic index compared to the control group. Low or high doses of Fe_2_O_3_-NPs administered to rats reduced (*p* < 0.001) final body weight and gonadosomatic index relative to the control group. In contrast, the RSV treatment restored (*p* < 0.001) the Fe_2_O_3_-NPs-induced reduction in final body weight and gonadosomatic index until they became non-significant relative to the control group.

### 3.2. Effect of RSV and/or Fe_2_O_3_-NPs on Spermiogram

As displayed in Figure 2B,C, the sperm motility percent, sperm concentration, and live sperm percentage were reduced (*p* < 0.001) in the low (38.37%, 20.17%, and 23.99%, respectively) and high (51.41%, 42.54%, and 61.49%, respectively) Fe_2_O_3_-NPs-exposed groups relative to the control group. The percent of sperm abnormalities, including detached and bifurcated sperm heads and short, coiled, and bent sperm tails, was increased (*p* < 0.001) by about twofold and threefold in the low and high Fe_2_O_3_-NPs-exposed groups, respectively, compared to the control group. In contrast, the sperm count, motility, and living sperm percentages were substantially restored by RSV oral dosing, but the sperm abnormality percentage decreased compared with Fe_2_O_3_-NPs-exposed rats. It is of note that all the spermiogram elements, except the sperm abnormalities, improved (*p* < 0.001) in the RSV+ Fe_2_O_3_-NPs-treated groups efficiently until they became not considerably different from those in the control group.

### 3.3. Effect of RSV and/or Fe_2_O_3_-NPs on Male Sex Hormones

**Figure 3:**
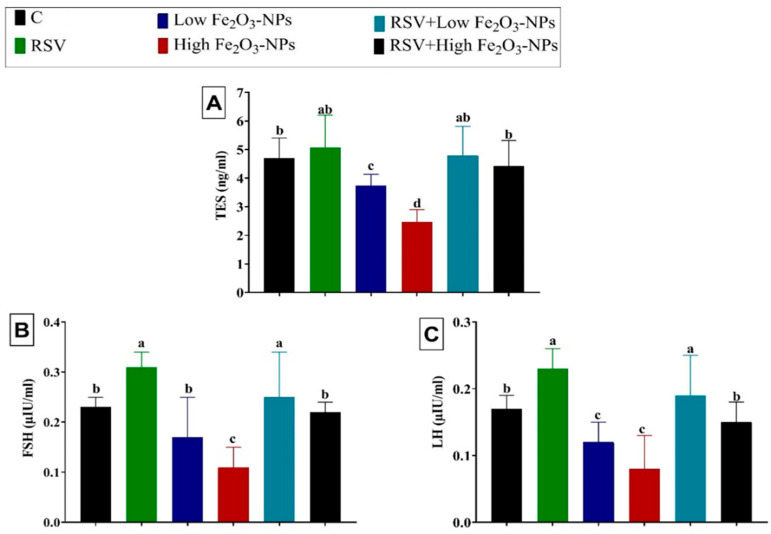
The effect of resveratrol (RSV; 20 mg/kg b.wt) and/or iron oxide nanoparticles (Fe_2_O_3_-NPs 3.5 or 7 mg/kg bwt) administration for eight weeks on the serum levels of male sexual hormones, including testosterone (TES) (**A**), follicle-stimulating hormone (FSH) (**B**), and luteinizing hormone (LH) (**C**) in male rats. Data expressed as mean ± SD, *n* = 9 for each group. Different letters (a, b, c, and d) on columns indicate statistically significant differences at *p* < 0.05 between the different experimental groups.

As demonstrated in Figure 3, the TES, FSH, and LH levels declined (*p* < 0.001) in the low (6.50%, 26.09%, and 29.41%, respectively) and high (38.25%, 52.17%, and 52.94%, respectively) Fe_2_O_3_-NPs-exposed groups compared to the control group. However, RSV oral dosing reestablished (*p* < 0.001) the Fe_2_O_3_-NPs-induced depletion in sexual hormone levels until it became not substantially different from the control group.

### 3.4. Effect of RSV and/or Fe_2_O_3_-NPs on Testicular Antioxidants and Lipid Peroxidation Level

**Figure 4:**
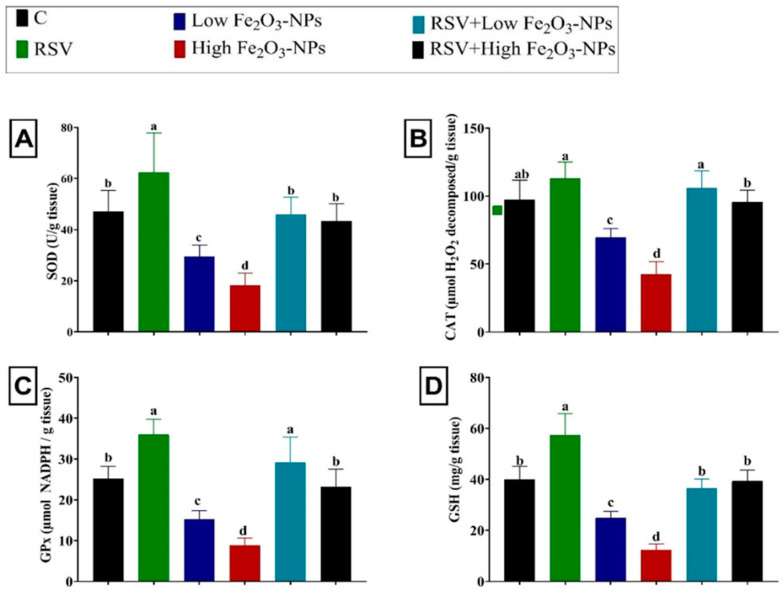
Effect of resveratrol (RSV; 20 mg/kg b.wt) and/or iron oxide nanoparticles (Fe_2_O_3_-NPs 3.5 or 7 mg/kg b.wt) administration for eight weeks on superoxide dismutase (SOD) (**A**) catalase (CAT) (**B**), glutathione peroxidase (GPx) (**C**), and reduced glutathione (GSH) (**D**) levels in the testicular tissues of male rats. Data expressed as mean ± SD, *n* = 9 for each group. Different letters (a, b, c, and d) on columns indicate statistically significant differences at *p* < 0.05 between the different experimental groups.

**Figure 5:**
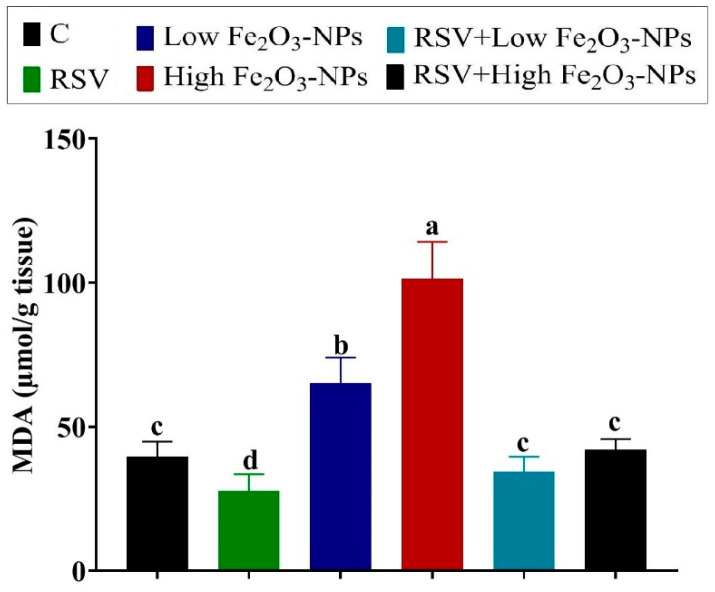
Effect of resveratrol (RSV; 20 mg/kg b.wt) and/or iron oxide nanoparticles (Fe_2_O_3_-NPs 3.5 or 7 mg/kg b.wt) administration for eight weeks on malondialdehyde (MDA) levels in the testicular tissues of male rats. Data expressed as mean ± SD, *n* = 9 for each group. Different letters (a, b, c, and d) on columns indicate statistically significant differences at *p* < 0.05 between the different experimental groups.

The noticeable depletion of the enzymatic antioxidants SOD (*p* = 0.012), CAT (*p* < 0.001), and GPx (*p* < 0.001) was recorded in the low (37.50%, 28.46%, and 39.37%, respectively) and high (61.31%, 56.30%, and 65.05%, respectively) Fe_2_O_3_-NPs-exposed groups relative to the control group (Figure 4). Furthermore, the non-enzymatic antioxidant content, GSH, was exhausted (*p* < 0.001) in the low and high Fe_2_O_3_-NPs-exposed groups (37.78% and 69.03%, respectively) relative to the control group. Nevertheless, compared to the control group, the level of lipid peroxidative marker (MDA) was increased (*p* < 0.001) by 64.61% and twofold in the low and high Fe_2_O_3_-NPs-exposed groups, respectively (Figure 5). However, in the testicular tissue of RSV+Fe_2_O_3_-NPs-treated rats, the CAT, SOD, GPx, and GSH levels were (*p* < 0.001) reestablished and MDA elevation was suppressed (*p* < 0.001) until it became not significantly different from the control group.

### 3.5. Effect of RSV and/or Fe_2_O_3_-NPs on Gene Expression in Testicular Tissue

**Figure 6:**
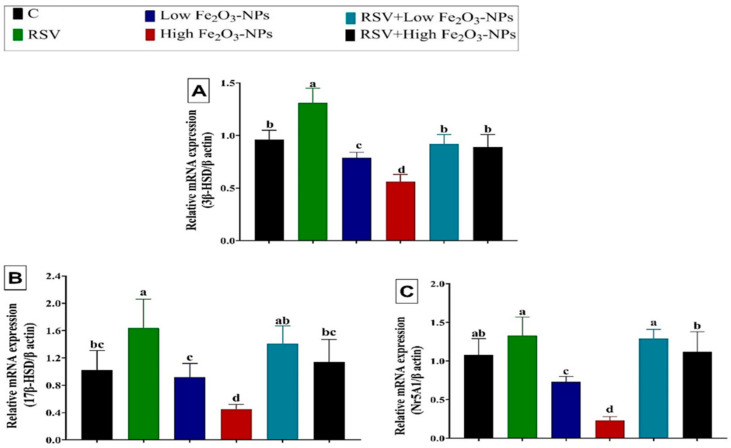
Effect of resveratrol (RSV; 20 mg/kg b.wt) and/or iron oxide nanoparticles (Fe_2_O_3_-NPs 3.5 or 7 mg/kg b.wt) administration for eight weeks on mRNA expression of (**A**) 3 beta-Hydroxysteroid dehydrogenase (*3β-HSD*), (**B**) 17-beta hydroxysteroid dehydrogenase (*17β-HSD*), and (**C**) Nuclear Receptor Subfamily 5 Group A Member 1 (*Nr5A1*) in the testicular tissues of male rats. Data expressed as mean ± SD, *n* = 9 for each group. Different letters (a, b, c, and d) on columns indicate statistically significant differences at *p* < 0.05 between the different experimental groups.

As displayed in Figure 6, steroidogenesis-related genes, including *3β-HSD*, *17β-HSD*, and *Nr5A1*, were downregulated (*p* < 0.001) in the testicular tissue of low (0.79 ± 0.03, 0.92 ± 0.08, and 0.73± 0.04, respectively) and high (0.56 ± 0.04, 0.45 ± 0.03, and 0.23 ± 0.03, respectively) Fe_2_O_3_-NPs-exposed rats relative to the control group (0.96 ± 0.05, 1.02 ± 0.12, and 1.08 ± 0.12, respectively). The RSV dosing to the low or high Fe_2_O_3_-NPs-exposed rats counteracted (*p* < 0.001) the downregulation of analyzed steroidogenesis-related genes until they became not significantly changed from the control group.

## 4. Discussion

Initially, in the current study, Fe_2_O_3_-NPs-exposed rats showed a significant decrease in final body weight and gonadosomtic index compared to the control group. In this context, Sundarraj et al. [17] reported that Fe_2_O_3_-NPs oral exposure (25 and 50 mg/kg) caused a significant decrease in food consumption, water intake, body weight gain, and organosomatic index. A similar reduction in body weight was recorded following exposure to other NPs, including silver NPs [63] and titanium dioxide NPs [64]. It is of note that in the current study, the gonadotropins level (LH and FSH) was significantly reduced despite this earlier reported inverse relationship between LH and FSH and body mass index changes [65]. Hence, despite the weight reduction, the recorded reduction of gonadotropins levels denotes that the Fe_2_O_3_-NPs exposure effect on the male sexual hormones and gonadosomatic index is a specific action rather than a non-specific stress effect. Fe_2_O_3_-NPs mediated testis weight decreases seemed to inhibit spermatogenesis after the germ cells’ death [66]. On the other hand, RSV oral dosing normalized reduced weight of the testes in Fe_2_O_3_-NPs-exposed rats possibly by cell stimulation and spermatogenesis due to RSV-mediated oxidative stress relief [67].

One key reason for the increase in male infertility is deteriorating semen quality [68]. Herein, the marked decline in sperm count, motility, and viability with a significant increase in morphological aberrations in the low or high Fe_2_O_3_-NPs-exposed rats reflected a pronounced deterioration of sperm quality. In this regard, Sundarraj et al. [17] reported that the fructose levels of the seminal vesicle decreased from Fe_2_O_3_-NPs. The decrease in the fructose amount in the seminal vesicle impedes energy in sperm [69] and contributes to seminal vesicle hypofunction that leads to infertility [70]. On the other hand, RSV treatment significantly improved the sperm quality in the low or high Fe_2_O_3_-NPs-exposed rats, denoting further improved fertility. Comparably, previous animal studies have shown that RSV successfully enhances the structure and quality of sperm as well as drugs/chemicals-induced testicular structural change, possibly due to its antioxidant properties [71,72,73,74].

The process of sperm production is known to be controlled by the three leading male fertility hormones: TES, FSH, and LH [75]. Our data revealed that Fe_2_O_3_-NPs exposure markedly decreased the male sexual hormones, including TES, FSH, and LH levels in the male rats. The former reductions could be linked to the earlier reported Fe_2_O_3_-NPs endocrine-disrupting activity that might have interrupted the hypothalamic-hypophyseal–testicular axis [15]. In addition, the Fe_2_O_3_-NPs-induced oxidative stress and lipid peroxidation could share in the reduction of male sexual hormones [76,77]. Similarly, other metal oxide NPs have been reported to reduce the three key male hormones levels and impair male fertility. For instance, the oral dosing of manganese oxides NP (100–400 mg/kg b.wt for 14 days) significantly reduced TES, FSH, and LH levels [78]. Interestingly, the re-establishment of the TES, FSH, and LH concentrations following RSV treatment displayed their protective effect against male sex hormones deficits mediated by Fe_2_O_3_-NPs exposure in the experimental animals. In this regard, Juan et al. [79] reported that RSV supplementation of normal rats led to increased hypothalamic–pituitary–gonadal axis function. Moreover, due to its structural similarity with estrogen, RSV is an estrogen agonist [80], which could promote the FSH and TES levels [79].

Diverse in vitro and in vivo studies have shown that multiple mechanisms could be involved in Fe_2_O_3_-NPs negative impacts, but the Fe_2_O_3_-NPs-mediated oxidative stress and lipid peroxidation is considered the dominant mechanism [8,81]. In the current investigation, the Fe_2_O_3_-NPs-exposed rats exhibited a marked reduction in enzymatic (CAT, GPx, and SOD) and non-enzymatic (GSH) antioxidants simultaneously with a sharp rise in the lipid peroxidation marker, MDA. SOD, CAT, GPx, and GSH are vital antioxidant molecules that guard against lipid peroxidation via ROS elimination. SOD catalyzes the dismutation of superoxide anion (O_2_^•^) radical to hydrogen peroxide (H_2_O_2_), whereas CAT degrades H_2_O_2_ into an oxygen molecule and a water molecule [82]. The H_2_O_2_ is also a substrate for GPx, which uses H_2_O_2_ to oxidize GSH [83]. The significant changes in this antioxidant system in the testes of rats exposed to Fe_2_O_3_-NPs concomitant with increased MDA levels allow us to conclude that the testicular antioxidant defenses did not protected the tissue from the action of Fe_2_O_3_-NPs-induced lipid peroxidation. Similarly, Sundarraj et al. [17] confirmed the reduction of GSH content in the male accessory organs on exposure to Fe_2_O_3_-NPs. Fe_2_O_3_-NPs in the cells remain in endosomes or lysosomes and become free iron, which raises the total iron pool within the cells [84]. Several previous studies have confirmed that iron, when present in excess within cells, interrupts redox homeostasis and catalyzes the ROS propagation, creating an oxidative environment [85,86]. By comparison, oral RSV dosing substantially restored the depleted antioxidant enzymes and suppressed the high MDA content of Fe_2_O_3_-NPs-exposed rats in their testicular tissue. RSV has been reported to cross the blood–testes barrier and give the testis its defensive effects [87]. The RSV-induced inhibition of oxidants in testicular tissue is probably linked to its capacity to trap superoxide anion, hydroxyl radicals, hydrogen peroxide, and up-control the production of endogenous antioxidants [88]. The RSV’s antioxidant activity is determined by the arrangement of functional groups on the nuclear structure. As a result, the configuration, substitution, and the total number of hydroxyl groups all significantly impact several antioxidant mechanisms, including radical scavenging and metal ion chelation. Previous research has shown that the hydroxyl group in the 4′ position is not the only determinant of antioxidant activity, but that the 3- and 5-OH groups also play a role [89,90]. The antioxidant effect of RSV on hydroxyl (•OH) and hydroperoxyl (•OOH) radicals were studied, and it was discovered that RSV could act as an efficient •OOH, and possibly •OOR, radical scavenger [91]. Additionally, the RSV antioxidant properties in vivo are more likely to be attributable to its effect as a gene regulator [92]. For instance, RSV inhibits NADPH oxidase-mediated release of ROS through downregulating the oxidase expression. Additionally, RSV reduces mitochondrial superoxide generation by motivating mitochondria biogenesis [93]. Furthermore, RSV increased the expression of various antioxidant enzymes in different cell types [94].

Testicular steroidogenesis is a regulated multistep process controlled by multiple genes [95,96]. TES is produced chiefly in Leydig cells through a series of enzymatic reactions. First, the steroidogenic acute regulatory (*StAR*) protein transfers cholesterol to the mitochondria [97]. Then, the mitochondrial cytochrome P450scc converts cholesterol into pregnenolone. Consequently, other enzymes (*3β-HSD*, *P450c17*, and *17β-HSD*) transform the pregnenolone into TES [98]. In addition, several transcription factors play essential roles in regulating steroidogenic genes. For instance, *Nr5A1* is a transcription factor that regulates the expression of steroidogenic genes like *3β-HSD* in Leydig cells. Hence, some authors explored the influence of metal oxide NPs on the expression of genes related to steroidogenesis [99]. For instance, Hussein et al. [100] reported that the intragastric administration of zinc oxide NPs (100 and 400 mg/kg b.wt) for 12 weeks significantly downregulated the expression of *17β-HSD*, *3β-HSD*, and *Nr5A1* and simultaneously reduced the TES level. Additionally, *3β-HSD* downregulation concomitantly with reduced TES level have been recorded with exposure to cerium oxide NPs (orally, 10–40 mg/kg b.wt, 32 days) in mice [98] and Fe_3_O_4_ (orally, 5 mg/kg b.wt, 79 days) in rats [101]. Consequently, in the current study, we assessed the expression of two steroidogenic genes, *3β-HSD*, and *17β-HSD* and the important regulating factor *Nr5A1*. The results revealed that the testicular *3β-HSD*, *17β-HSD*, and *Nr5A*1 mRNA expressions were downregulated in the Fe_2_O_3_-NPs-exposed rats demonstrating reduced testicular steroidogenesis. Thus, we hypothesize that suppression of the rate-limiting step of steroidogenesis and decreased transcript levels of genes encoding for *3β-HSD*, and *17β-HSD*, which catalyze the downstream reactions in steroidogenesis, are responsible for the decreased TES levels and the subsequent LH and FSH reduction in the present study. The decline of the three male sex hormone levels was reflected in the decrease in sperm count and motility in Fe_2_O_3_-NPs-exposed rats. Comparably, the downregulation of steroidogenesis-related genes was the machinery mechanism of the testicular damage induced by several metal NPs [98,101].

Accumulative evidence has shown that testicular steroidogenesis dysfunction and spermatogenesis triggered by chemical-induced oxidative stress were related to impaired male fertility [102]. Additionally, many studies suggested that testicular steroidogenesis is interrupted in the oxidative stress conditions via the inhibition of the major transcription factors that regulate the expression of steroidogenic enzyme genes [103,104,105,106,107]. Moreover, a strong correlation has been reported between steroidogenesis disruption and lipid peroxidation [108,109]. Hence, the Fe_2_O_3_-NPs-induced oxidative stress and lipid peroxidative damage in the testicular tissues could effectively impair the steroidogenesis process. However, further studies are highly recommended on the underlying mechanisms of Fe_2_O_3_-NPs-induced testicular damage, focusing on the protein expressions of the tested genes and other genes involved in the steroidogenesis process.

On the contrary, a considerable recovery was distinguished in the steroidogenic-related gene expression following the RSV oral dosing. The potent antioxidant activity and anti-lipid peroxidative action of RSV could be the initial mechanism through which the other beneficial effects, including the correction of the transcription of steroidogenic-related genes, have been attained. In accordance with our results, Banerjee et al. [110] reported that RSV (oral doses of 50 mg/kg b.wt daily for 60 days) effectively mitigated oxidative stress in Leydig cells and increased the transcriptional level of *CYP11A1*, *17β-HSD*, *3β-HSD*, and *StAR* and thus protected steroidogenesis from Benzo(a)pyrene’s negative impact in rats.

Despite the evident protective role of RSV against the Fe_2_O_3_-NPs-induced impairment in sperm quality, male hormone balance, and the regulation of some steroidogenic-related genes, the present study has some limitations. First, several probable underlying mechanisms have not been covered, such as the regulation of the apoptotic events, the DNA damage repair, and the inflammatory events. Second, we did not experimentally examine the molecular effects of Fe_2_O_3_-NPs and RSV on the specific testicular cell types. Third, the expressions of other genes incorporated into the pathway regulation of testicular steroidogenesis have not been explored. Therefore, the current study findings need to be deepened by future mechanistic studies to cover the gap in knowledge regarding the other probable underlying mechanisms of both RSV and Fe_2_O_3_-NPs on male fertility.

## 5. Conclusions

The findings of the current study provide novel clues for the possible involvement of the downregulation of steroidogenesis-related genes (*3β-HSD*, *17β-HSD*, and *Nr5A1*) in the induction of testicular damage in Fe_2_O_3_-NPs-exposed rats. Our results suggested that the RSV administration could be beneficial in the defense against Fe_2_O_3_-NPs-induced impaired sperm quality and testicular damage via amplifying the antioxidant capacity and the subsequent appropriate steroidogenesis-related gene regulation. Thus, RSV could be suggested as a natural dietary supplement in persons frequently exposed to Fe_2_O_3_-NPs. Further studies are highly recommended on the other probable mechanisms implicated in the protective role of RSV against chemical-induced testicular injury, such as anti-apoptotic and anti-inflammatory effects, as well as the repair of DNA damage.

## Figures and Tables

**Table 1:** Primer sequences, accession number, and product size for the quantitative RT-PCR for the analyzed genes in the testicular tissue.

Gene		Primer Sequences	Reaction Conditions	Accession No.	PCR Product Size
*3β-HSD*	**F**	**5′**–GCATTAACCCCACTCCCACT–**3′**	95 °C, 10 min/60 °C, 30 s/72 °C, 5 min (35 cycles)	NM 017265	146 bp
	**R**	**5′**–GGACCCTGACCTCCTTCAGA–**3′**			
*17β-HSD*	**F**	**5′**–GTGTGCACATTTTCCAAGGC–**3′**	95 °C, 10 min/60 °C, 30 s/72 °C, 5 min (35 cycles)	NM 054007	144 bp
	**R**	**5′**–TTTAACAAACTCATCGGCGG–**3′**			
*Nr5A1*	**F**	**5′**–CGCCAGGAGTTTGTCTGTCT–**3′**	95 °C, 10 min/60 °C, 30 s/72 °C, 5 min (35 cycles)	NM 001191099	185 bp
	**R**	**5′**–ACCTCCACCAGGCACAATAG–**3′**			
*β-actin*	**F**	**5′**–CCTGCTTGCTGATCCACA–**3′**	95 °C, 10 min/60 °C, 30 s/72 °C, 5 min (35 cycles)	V01217	97 bp
	**R**	**5′**–CTGACCGAGCGTGGCTAC–**3′**			

F indicates forward primer; R indicates reverse primer; *3β-HSD*: 3 beta-Hydroxysteroid dehydrogenase; *17β-HSD*: 17-beta hydroxysteroid dehydrogenase; *Nr5A1*: Nuclear Receptor Subfamily 5 Group A Member 1.

## Data Availability

Data is contained within the article.
